# Stimulation of the autonomic nervous system in colorectal surgery: a study protocol for a randomized controlled trial

**DOI:** 10.1186/1745-6215-13-93

**Published:** 2012-06-27

**Authors:** Tim MP Berghmans, Karel WE Hulsewé, Wim A Buurman, Misha DP Luyer

**Affiliations:** 1 Faculty of Medicine, Maastricht University, Maastricht, ER 6229, The Netherlands; 2 Department of Surgery, Orbis Medical Centre, Sittard, MB 6130, The Netherlands; 3 Department of Surgery, Maastricht University, Maastricht, ER 6229, The Netherlands; 4 Department of Surgery, Catherina Hospital Eindhoven, PO Box 1350, 5602 ZA, Eindhoven, The Netherlands

**Keywords:** Postoperative ileus, Chewing gum, Inflammation, Colorectal surgery, Autonomic nervous system

## Abstract

**Background:**

Postoperative ileus (POI) is a well-known complication of abdominal surgery and is considered to be caused by a local inflammation in the gut. Previously it has been shown that both local and systemic inflammation can be reduced by stimulation of the autonomic nervous system via lipid rich nutrition. Stimulation of the autonomic nervous system releases acetylcholine from efferent vagal nerve endings that binds to nicotinic receptors located on the inflammatory cells leading to a decrease of pro-inflammatory mediators. Besides administration of nutrition there are other ways of stimulating the autonomic nervous system such as gum chewing.

**Methods/design:**

This prospective, placebo-controlled randomized trial will include 120 patients undergoing colorectal surgery which are randomized for gum chewing preoperatively and in the direct postoperative phase or a placebo. Postoperative ileus will be assessed both clinically by time to first flatus and time to first defecation and by determination of gastric motility using ultrasound to measure dimensions of the antrum. Furthermore the inflammatory response is quantified by analyzing pro-inflammatory mediators. Finally, markers of gut barrier integrity will be measured as well as occurrence of postoperative complications.

**Discussion:**

We hypothesize that chewing gum preoperatively and in the direct postoperative phase in patients undergoing colorectal surgery dampens local and systematic inflammation, via activation of the autonomic nervous system. Down-regulation of the inflammatory cascade via stimulation of the vagus nerve will ameleriote POI and enhance postoperative recovery.

**Trial registration:**

NTR2867

## Background

All intra-abdominal surgical procedures, even minimally invasive, are followed by a transient episode of gastrointestinal hypomotility making postoperative ileus (POI) a common clinical phenomenon after abdominal surgery. Therefore it has been suggested to accept POI as an obligatory physiological response of the intestine when performing abdominal surgery [1]. However, POI clearly has a significant impact on patient morbidity and results in a prolonged hospital stay and significantly contributes to annual healthcare costs [1]. Implementation of fast-track colorectal surgical programs have already shown promising results in reducing overall hospital stay by epidural analgesia, earlier nutrition, and mobilization after surgery [2]. Further reduction of this postoperative transient episode of gastrointestinal hypomotility will add to the enhanced recovery after colorectal surgery.

Research has shown that the extent of POI is influenced by the degree of surgical trauma, bowel manipulation and duration of surgery [3]. The inflammatory response is regarded to play a key role in the etiology of POI next to factors as sympathetic neural reflexes and release of hormones and neurotransmitters. It is thought that handling of the intestine during abdominal surgery triggers the inflammatory cascade leading to local inflammation in the intestinal muscular layer and influx of leucocytes [4]. This local inflammation is important in the development of gastrointestinal hypomotility after abdominal surgery and thus POI [1,4,5].

Previous studies have shown that vagus nerve stimulation ameliorates the local and systemic inflammatory response by binding of acetylcholine to α7 nicotinic ACh receptors (nAChrR) expressed on macrophages resulting in down-regulation of tissue macrophage reactivity and cytokine release [6,7]. This neural feedback loop can be triggered mechanically, pharmacologically, or in a physiological way via administration of enteral nutrition leading to an instant reduction of the inflammatory cascade. In experimental rat models we have previously demonstrated that preoperative administration of lipid-enriched enteral nutrition strongly attenuates the inflammatory response and reduces POI via release of cholecystokinin and activation of afferent vagal fibers [8,9]. However, clinical research is necessary to substantiate these effects in humans.

Activation of the autonomic nervous system can also be established by sham-feeding such as chewing gum [10-12]. Several studies have shown favorable effects of chewing gum after surgery for POI, measured as time to flatus and overall hospital stay [13-15]. However, the underlying mechanism remains unclear. Furthermore, based on experimental data previously referred to, chewing gum before the surgery would be expected to be most effective whereas. However, until now all studies administered chewing gum in the postoperative phase only.

In this study we investigate the effect of perioperative gum chewing on postoperative inflammatory mediators and evaluate clinical parameters such as POI in patients undergoing colorectal surgery.

### Study objectives

The primary objective of this study is to determine the effects of gum chewing in the perioperative phase on the extent of POI, overall hospital stay, and morbidity and mortality. The secondary objective is to determine the effects of gum chewing in the perioperative phase on postoperative inflammation and tissue damage.

### Methods and design

This trial is designed as a prospective placebo-controlled randomized trial in which the effect of chewing gum before and directly after colorectal surgery is compared to a placebo.

### Study population

Patients older than 18 years of age undergoing elective colorectal surgery are eligible for inclusion. In total 120 patients will be included. Patients are excluded in case of peritoneal carcinomatosis, inflammatory bowel disease, previous gastric or esophageal surgery (due to vagus nerve injury), a pre-existent ileostoma, allergy for mint, or gut motility influencing agents (for example, tricyclic antidepressants). Furthermore, patients with disturbance of the acetylcholine metabolism in any way, such as neurological diseases, medication (SSRI), or depression will be excluded.

### Participating centers

Patients eligible for enrolment will be included in two hospitals in the Netherlands; Orbis Medical Center in Sittard and Catharina Hospital Eindhoven.

### Randomization

After obtaining written informed consent patients will be randomly assigned to one of two groups by randomization software (TenAlea software adapted for minimization randomization). Based on this output a sequentially numbered, opaque, sealed envelope will be opened by which the allocation is revealed. Patients are either allocated to the experimental group and receive chewing-gum preoperatively and directly postoperative until start of enteral nutrition. The control group receives a placebo (dermal patch) for the same period, which does not contain any active ingredient, although patients are told otherwise. The dermal patch was chosen as placebo since mimicking the chewing of chewing gum in any other way would function as sham-feeding and thereby contaminate the results. The dermal patch can be considered comfortable, easy to use, and non-invasive, and serves the placebo purpose of patients assuming the patch to contain an active ingredient. Opening of the sealed envelopes and the administration of the intervention or the placebo is performed by nursing staff not participating in this trial. Ethical approval for this study was granted by the Medical Ethics Committee Atrium-Orbis-Zuyd (Heerlen, the Netherlands).

### Study outline

All subjects are allowed to drink clear fluids up to 2 h before surgery. Patients allocated to the experimental group start chewing gum 3 h preoperatively until surgery, 3 h postoperatively gum chewing is continued. Though patients are free to chew the gum to their own liking during these periods, they are being encouraged to chew the gum as often as possible. Gum chewing is discontinued at the moment at which normal solid food intake is being tolerated. The control group will receive a dermal patch at the same moments as the experimental group and are instructed not to chew any gum pre- or postoperatively. The patch will be placed at a standardized location in the lumbar region. Both groups receive the same fast-track surgical protocol as any other patients undergoing colorectal surgery in our center. At moment of inclusion, consent is obtained and patient information will be given in which both the gum chewing as the placebo (dermal patch) are described as being possibly effective. The expected effective intervention (gum chewing) and definitive conclusion will not be revealed until completion of the trial.

Blood and urine samples will be collected at several predefined moments in relation to the incision time of the procedure. Tissue samples from the resected colorectal segment are collected during the surgical procedure for immunohistochemistry, Western blot, and polymerase chain reaction (PCR) analysis. When collected all samples are stored at −80 °C until further analysis. The inflammatory response is measured by analyzing the blood plasma and tissue samples for inflammatory mediators (amongst which TNF-alpha and interleukins such as IL-6 and IL-8). Intestinal tissue damage is measured by analysis of several markers for intestinal damage in blood and tissue samples such as intestinal fatty acid binding protein (I-FABP).

Patient characteristics and clinical parameters, such as vomiting and time to return of a normal gastrointestinal transit, are registered in an electronic database.

On admission, a quality of life checklist (EORTC QLQ C-30) for cancer patients is filled out, which is repeated at time of discharge. Patients are ready for discharge according to known criteria as in fast-track surgery [16,17]. At the second postoperative day gastrointestinal transit is assessed by ultrasonography by a standard protocol.

### Antrum measurements

POI is often measured clinically based on parameters such a time to first flatus and first defecation. These are more or less subjective parameters of gastric motility [14]. To provide an objective and precise parameter for gastric motility an ultrasound of the gastric antrum is performed in the evening of the second postoperative day. Previous studies have already shown antrum measurements to be representative for determining gastric emptying and thereby gastric motility [18-20]. In short, subjects receive a standardized meal after which antrum measurements are done. All results of these measurements are registered in an electronic database.

### Statistical analysis

Sample size is calculated with a Power analysis and was aimed at POI and length of stay. We considered a decrease of 1 day of admission from the average 5 days for current fast-track protocols in our center to be clinically relevant. To achieve a power of 0.9 with an alpha of 0.05 and a beta of 0.1 for a decrease of 1 day of the mean length of stay each group needs to consist of 60 subjects when performing a unpaired *T*-test. The analysis will be done according to the intention-to-treat principle. When using POI as primary outcome parameter for the Power analysis slightly fewer patients would be required per group due to the larger effect size. With the current sample size, both parameters are adequately powered.

## Discussion

POI is defined as delayed return of gastrointestinal motility occurring after abdominal surgical procedures. Recent advancements in perioperative care significantly enhanced patient recovery and reduced the overall hospital stay. However POI remains an important clinical problem without a therapeutic solution and interventions rely primarily on supportive measures [9,21].

Furthermore, POI also contributes to a prolonged length of stay that leads to increased healthcare costs [22]. The annual costs related to POI have been estimated to be as much as $1.47 billion annually in the US [1,22]. Reducing POI and thereby diminishing length of stay will result in a significant decrease of healthcare costs.

It is thought that the inflammatory response within the muscular layer of the gut which is triggered by intestinal manipulation plays a key role in the etiology of POI. Reduction of this postoperative transient episode of hypomotility of the gastrointestinal tract by dampening the inflammatory response seems a promising approach in ameliorating POI. We have already demonstrated that administration of enteral nutrition dampens the inflammatory response in experimental models [8]. This positive feedback mechanism involves release of cholecystokinin leading to activation of the autonomic nervous system. Sham-feeding is a potential other way of stimulating the autonomic nervous system after abdominal surgery.

Sham-feeding by chewing gum has been shown to ameliorate POI following gastrointestinal surgery when started after surgery [13-15]. However, the mechanism underlying the beneficial effect of gum chewing on POI remains elusive. Based on experimental studies, it may well be that activation of the autonomic nervous system leading to an inhibition of the inflammatory response plays an important role. In this light, chewing gum before the surgery would be expected to be most effective.

Therefore, our hypothesis is that chewing gum before and directly after colorectal surgery will dampen the inflammatory response, both locally and systematically, via stimulation of afferent vagal nerve fibers. Hereby, POI will be ameliorated and patients’ recovery will be enhanced.

This study was designed to investigate the effect of chewing gum on POI, the inflammatory response, and length of stay.

## Trial status

Ongoing trial, patient recruitment.

## Abbreviations

POI, Postoperative ileus.

## Competing interests

The authors declare that they have no competing interests.

## Authors’ contribution

TB drafted the manuscript. ML authored the writing of the manuscript. All authors participated in the design of the study and are local investigators at the participating centers. All authors read, edited, and approved the final manuscript.
